# Genome-wide support for incipient Tula hantavirus species within a single rodent host lineage

**DOI:** 10.1093/ve/veae002

**Published:** 2024-01-05

**Authors:** Anton Labutin, Gerald Heckel

**Affiliations:** Institute of Ecology and Evolution, University of Bern, Baltzerstrasse 6, Bern 3012, Switzerland; Institute of Ecology and Evolution, University of Bern, Baltzerstrasse 6, Bern 3012, Switzerland

**Keywords:** host-pathogen co-evolution, *Microtus arvalis*, common vole, virus speciation, genomic admixture, RNA virus

## Abstract

Evolutionary divergence of viruses is most commonly driven by co-divergence with their hosts or through isolation of transmission after host shifts. It remains mostly unknown, however, whether divergent phylogenetic clades within named virus species represent functionally equivalent byproducts of high evolutionary rates or rather incipient virus species. Here, we test these alternatives with genomic data from two widespread phylogenetic clades in *Tula orthohantavirus* (TULV) within a single evolutionary lineage of their natural rodent host, the common vole *Microtus arvalis*. We examined voles from forty-two locations in the contact region between clades for TULV infection by reverse transcription (RT)-PCR. Sequencing yielded twenty-three TULV Central North and twenty-one TULV Central South genomes, which differed by 14.9–18.5 per cent at the nucleotide and 2.2–3.7 per cent at the amino acid (AA) level without evidence of recombination or reassortment between clades. Geographic cline analyses demonstrated an abrupt (<1 km wide) transition between the parapatric TULV clades in continuous landscape. This transition was located within the Central mitochondrial lineage of *M. arvalis*, and genomic single nucleotide polymorphisms showed gradual mixing of host populations across it. Genomic differentiation of hosts was much weaker across the TULV Central North to South transition than across the nearby hybrid zone between two evolutionary lineages in the host. We suggest that these parapatric TULV clades represent functionally distinct, incipient species, which are likely differently affected by genetic polymorphisms in the host. This highlights the potential of natural viral contact zones as systems for investigating the genetic and evolutionary factors enabling or restricting the transmission of RNA viruses.

## Introduction

The evolution of parasites is usually tightly linked to their hosts, driven to a large degree by the parasites’ total loss of fitness when they fail to infect their host species (Vale and Little 2009; Penczykowski, Laine, and Koskella 2016; Simmonds, Aiewsakun, and Katzourakis 2019; Ebert and Fields 2020). Similarity in physiological features, such as in the immune system, affects the range of potential host species, and thus, there is often a strong phylogenetic component in host–parasite relationships (Clark et al. 2018; Mollentze et al. 2020). Acellular parasites (viruses and bacteriophages) are particularly affected by incompatibilities with their hosts, as they lack independent metabolisms and require tight interaction with the host’s cellular machinery for successful reproduction and transmission (Simmonds, Aiewsakun, and Katzourakis 2019). As a result, functional diversification and speciation in viruses depend on the genetic environment encountered in their hosts, with host–virus co-divergence (Switzer et al. 2005; Rector et al. 2007) and host switches (Parrish et al. 2008; Lin et al. 2012; Mélade et al. 2016) being the most common drivers of viral speciation.

Similar to the variety of definitions of species in their eukaryotic hosts (e.g. Galtier 2019; Kollár, Poulíčková, and Dvořák 2022), there is no universal consensus on the definition of virus species, and thus, their diversity is difficult to estimate. The most commonly accepted set of definitions of virus species is provided by the International Committee on Taxonomy of Viruses (ICTV). However, these definitions vary for each virus family and distinguish virus species based on a combination of factors, such as ‘natural and experimental host range, cell and tissue tropism, pathogenicity, vector specificity, antigenicity, and the degree of relatedness of their genomes or genes’ (International Committee on Taxonomy of Viruses (ICTV) 2021). Depending on the particular case, these criteria are subject to discussion and carry a level of ambiguity between many viral families (Adams et al. 2013; Van Regenmortel et al. 2013; Shapiro and Polz 2015). The criteria of the ICTV are not uniformly applied across all virus families, and as a result, viral species designations tend to lack a universal biological foundation (Simmonds et al. 2017).

The definitions of the ICTV are well suited for identifying deeply separated virus species, but were not intended to classify virus taxa that have not reached the threshold levels of evolutionary divergence but show already functionally relevant differences acquired in the process of speciation analogous to incipient eukaryotic species. A simplified species concept for acellular organisms has been proposed recently based on rates of gene flow (Bobay and Ochman 2018). This concept establishes species boundaries between virus populations in contact with one another that show no signs of gene exchange, i.e. reassortment and recombination. In this study, we use this definition to distinguish incipient virus species, although it has some limitations. Viruses with only a single segment are unable to reassort, which restricts their potential for genomic exchange to recombination. Furthermore, suitable conditions for potential co-infections by different virus clades need to exist, i.e. there should be no ecological barrier to reassortment and recombination.

Many named virus species are subdivided into deeply diverged phylogenetic clades, which can be spatially associated with the distribution of closely related host species (e.g. de Bellocq et al. 2015) or evolutionary lineages within the host (Saxenhofer et al. 2019). These clades could indicate speciation events within the virus cryptic to current taxonomy. Polymorphisms at relatively few host loci may be decisive for functional virus divergence (Meyer et al. 2016), and even single host genes have been shown to restrict the range of species that a virus can infect (Stremlau et al. 2004; van Doremalen et al. 2014; Long et al. 2016). Thus, viral clades that are confined to parapatric distribution ranges within a single evolutionary host lineage could actually represent distinct evolutionary units. These may result from cryptic or incipient speciation events on the virus side related to few genetic host polymorphisms (Saxenhofer et al. 2022). Parapatric distribution ranges have been detected at many levels of virus evolution, but further inferences are often limited because the geographic scale analysed is typically very coarse compared to the dispersal ability of the natural hosts and genetic resolution is often lacking (e.g. Drewes et al. 2017; Jeske et al. 2019).

Here, we investigated potential incipient speciation between two clades of Tula hantavirus (TULV) within its reservoir host, the common vole (*Microtus arvalis*). The genus *Orthohantavirus* contains currently thirty-eight virus species recognized by ICTV (Walker et al. 2020), which have often been linked to a single reservoir host species each. Both long-term co-speciation and occasional host shifts have been shown to play an extensive role in the deeper evolutionary history of hantaviruses (Guo et al. 2013). Recently, the ICTV proposed a new definition for classifying hantavirus species, setting the cut-off at a pairwise-evolutionary distance (PED) of 0.1 in DEmARC analysis for separate species (Laenen et al. 2019; Kuhn et al. 2023), which is likely to affect the species number once implemented.

TULV, like all hantaviruses, is a three-segmented, negative-strand RNA virus (Vaheri et al. 2013). It is horizontally transmitted in its rodent reservoir hosts causing chronic, asymptomatic infections (Forbes, Sironen, and Plyusnin 2018). The distribution of deep phylogenetic clades in TULV in Europe is partly associated with morphologically cryptic evolutionary lineages in its reservoir host, which suggests the potential importance of co-divergence in this system (Heckel et al. 2005; Saxenhofer et al. 2019; Schmidt et al. 2016; see later). Detailed analyses of the contact region between the TULV clades Central South (TULV-CEN.S) and Eastern South (TULV-EST.S) revealed an extremely tight association with the Central and Eastern evolutionary lineages in *M. arvalis* (Saxenhofer et al. 2019). These TULV clades occupy non-overlapping distribution ranges despite frequent dispersal and local gene flow between their host lineages. Their adaptive divergence and functional separation likely evolved only after a spillover event across the host hybrid zone (Saxenhofer et al. 2019). Further analyses identified a relatively small number of host genes that may contribute to limiting the effective transmission of these two TULV clades across the hybrid zone between host lineages (Saxenhofer et al. 2022).

**Figure 1.**
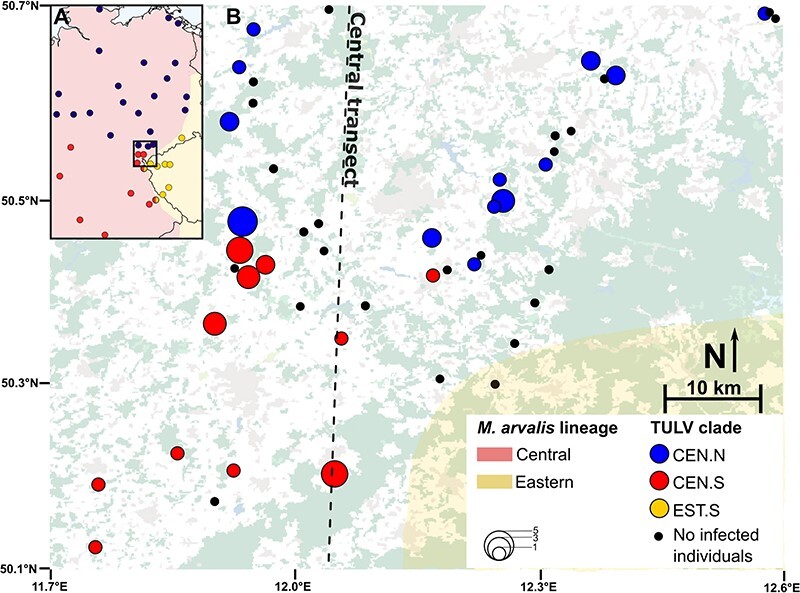
Contact area between Central European TULV clades in their natural rodent host. (A) Overview of our study area (square) in eastern Germany and the western Czech Republic with the evolutionary lineages Central (red shaded) and Eastern (yellow shaded) in the common vole (*M. arvalis*). The distribution of the TULV clades TULV-CEN.N (blue), TULV-CEN.S (red), and TULV.EST.S (yellow) is shown across Central Europe (based on Schmidt et al. 2016; Saxenhofer et al. 2019). (B) Sampling sites of common voles across the Central transect. Coloring of populations with infected individuals corresponds to (A). Circle sizes correspond to the frequency of infected individuals. Black dots indicate sampling sites where no infected individuals were detected. The area shaded in yellow indicates the extent of the Eastern *M. arvalis* lineage, as also indicated in (A) (Saxenhofer et al. 2019; 2022). The dashed line indicates the axis of the Central transect. The background map shows bodies of water in blue, settlements in grey, forests in green, and fields in white.

In this study, we use this virus–host system to test whether two deeply diverged phylogenetic clades of TULV within a single evolutionary lineage of *M. arvalis* show spatial and genomic properties consistent with distinct ‘biological species’ *sensu*  Bobay and Ochman (2018). We build on phylogeographic data of partial TULV sequences, which indicated that the TULV clades Central North (TULV-CEN.N) and Central South (TULV-CEN.S) have parapatric distribution ranges within the central mitochondrial lineage of *M. arvalis*, which occupies large areas of Central Europe (Fig. 1; Schmidt et al. 2016). We combined fine-scale sampling of the potential contact region with genome-wide sequence data of the virus and the host populations in order to determine the extent of evolutionary divergence across the sampling area. If the level of functional divergence between the clades matched or exceeded TULV-CEN.S and TULV-EST.S from Saxenhofer et al. (2019), we would expect to find a sharp transition without genetic exchange between the clades despite their genetically similar hosts.

## Materials and Methods

### Sampling

We sampled common voles (*M. arvalis*) at forty-five trapping locations in Eastern Germany (Fig. 1) where we expected the contact between TULV-CEN.N to the north and TULV-CEN.S to the south based on Schmidt et al. (2016) and Saxenhofer et al. (2019). We termed this sampling area the ‘Central transect’ because the TULV contact was expected within the Central common vole lineage. Southernmost sampling locations from the Central transect partially overlapped with the Porcelain transect from Saxenhofer et al. (2019); 2022). Rodent trapping was performed after ethical evaluation and approval by the Bernese cantonal commission on animal experimentation under permits BE-33/14 and BE-86/17. Common voles were trapped using snap traps and stored at −20°C immediately after collection. An overview over all samples analysed in this study can be found in Supplementary Table S1.

### TULV screening, whole-genome sequencing, and phylogeny

We screened 247 adult common voles from forty-two sampling sites for TULV infections. Screening was only performed for adult voles with a body weight of at least 20 g because infection rates of lighter animals are extremely low (<0.5 per cent; Schmidt et al. 2021; unpublished data) and juveniles of infected mothers are protected by maternal antibodies (Kallio et al. 2006). We extracted RNA from lung tissue with a modified QIAzol protocol (Schmidt et al. 2016). TULV infections were detected by PCR amplification of a fragment of the nucleocapsid gene in the S-segment of TULV using the RT-PCR assay detailed in Essbauer et al. (2006) and gel electrophoresis. Library preparation, sequencing, and genome assembly followed the hybrid sequence capture protocol in Hiltbrunner and Heckel (2020). We prepared RNA libraries for all TULV-infected vole hosts, as well as five additional TULV samples from sites in the western end of the Porcelain transect from Saxenhofer et al. (2019). These samples constituted four novel genomes and one replicate TULV genome (MarDHg01) originally obtained with shotgun sequencing and comparatively low read depth in Saxenhofer et al. (2019). Two novel samples failed library quality controls and were not included in further downstream processing.

Custom baits from Hiltbrunner and Heckel (2020) were used to capture and enrich viral sequences in libraries, which were then sequenced on a MiSeq (Illumina, San Diego, CA, USA) with 2× 300 cycles on the Next-Generation Sequencing Platform of the University of Bern. We used the Iterative Virus Assembler (Hunt et al. 2015) for *de novo* assembly of TULV genomes, which were then reference mapped against the viral consensus genomes for quality control and inference of mapping statistics (see details in Hiltbrunner and Heckel 2020). For two TULV genomes, missing information for nucleotides in the L-segment (four positions in MarDOk02 and thirty-one in MarDNk29) was imputed based on the sequence of the closest genetic relative from the same sampling site (Saxenhofer et al. 2019). For each genome, we calculated the proportion of sites with a read depth of at least 3× and 20×, respectively, as well as the average genomic coverage using R.

### TULV phylogenetic analysis

Phylogenetic analysis and clade assignment of the TULV genomes were based on the coding nucleotide sequences (CDSs) and the derived AA sequences of the viral genomes. For the CDS, both the concatenated sequence of all segments and individual segments were analysed. We used published TULV genomes with a complete CDS (excluding genomes with missing sites or sections of the CDS) from Central Europe (Kukkonen, Vaheri, and Plyusnin 1998; Saxenhofer et al. 2019; Hiltbrunner and Heckel 2020) as the reference. In addition, four published TULV genomes from *Microtus obscurus* from China (Chen et al. 2019) and two published *Puumala orthohantavirus* genomes (Vapalahti et al. 1992; Piiparinen et al. 1997; Ali et al. 2015) were included as outgroups (Supplementary Table S2).

The phylogenetic analysis of nucleotide sequences was performed using MrBayes version 3.2.7a (Ronquist et al. 2012) on the CIPRES platform (Miller, Pfeiffer, and Schwartz 2010). We performed Markov chain Monte Carlo (MCMC) sampling for up to 10^8^ generations in four independent runs comprising four chains, implementing reversible-jump sampling over the entire general time-reversible substitution model space (Huelsenbeck, Larget, and Alfaro 2004). After discarding a burn-in fraction of 25 per cent, samples were recorded every 10^3^ generations. Chains converged after 155,000 generations. The phylogenetic analysis for AA sequences was performed in MEGA X (Kumar et al. 2018). Tree topology was inferred using the maximum-likelihood method based on the Jones-Taylor-Thornton (JTT) matrix–based model (Jones, Taylor, and Thornton 1992) with 1,000 bootstraps. The final tree was obtained by applying neighbour-joining and BioNJ algorithms to a matrix of pairwise distances estimated using the JTT model and then selecting the topology with superior log-likelihood value. Phylogenetic trees were drawn and edited using the online platform iTOL v5 (Letunic and Bork 2019). Maps visualizing the viral clade distribution were created in R using the geosphere package (Hijmans et al. 2017), and topographic backgrounds are based on Globeland30 (Jun, Ban, and Li 2014).

### TULV sequence diversity and signatures of selection

**Figure 2.**
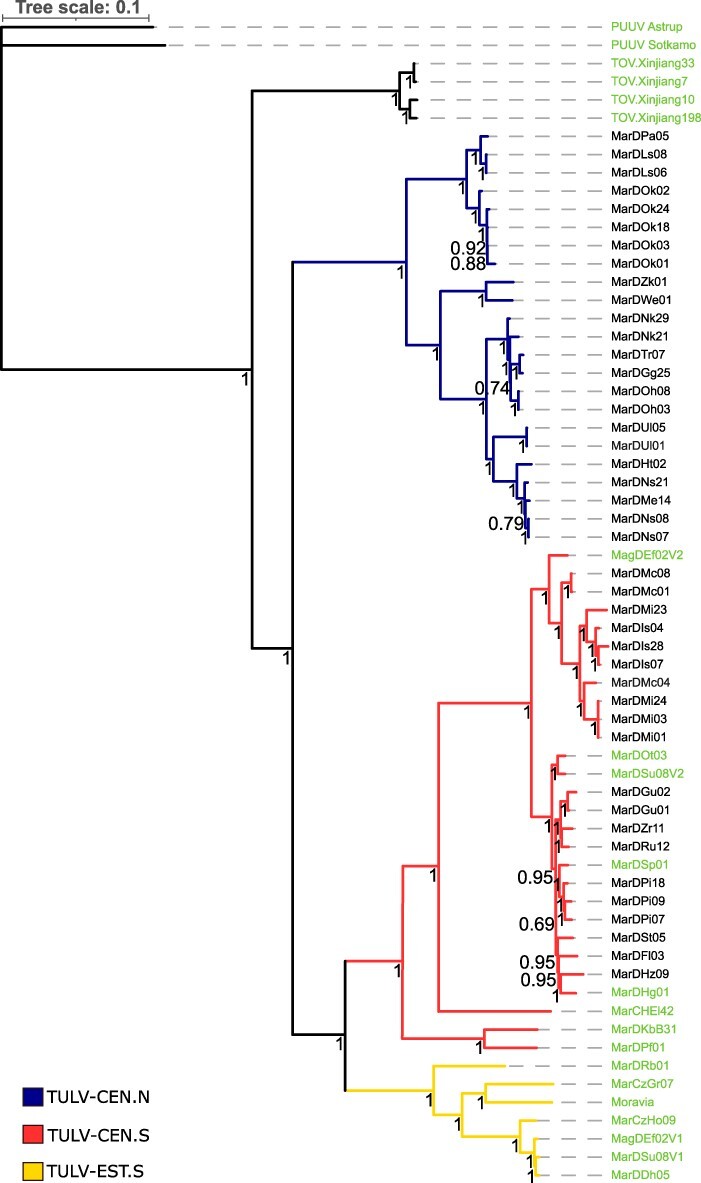
Phylogenetic relationships of TULV genomes from Central Europe with complete coding sequences. Phylogenetic analysis was based on the concatenated complete coding regions of TULV with samples from Xinjiang (China) and *Puumala orthohantavirus* as outgroups. Names in black show new TULV genome sequences from this study, while green represents reference and outgroup sequences. Bayesian posterior probabilities are included for all nodes. The scale bar on top shows evolutionary distance in substitutions per nucleotide.

We used DnaSP version 5 (Rozas et al. 2003) to estimate genome-wide nucleotide diversity and between-clade divergence and perform sliding-window analyses (window size = 30 and step size = 10) of *d*_N_/*d*_S_ ratios (ratio of non-synonymous to synonymous substitutions) and *D*_XY_ (average number of nucleotide substitutions per site) across the CDS of all three TULV segments. AA divergence was estimated in MEGA X by calculating the mean *p*-distance within and between TULV clades. Additionally, PEDs were also calculated using Tree-Puzzle version 5.2, using a maximum-likelihood approach with a Whelan and Goldman substitution model, analogous to the DEmARC analysis in Laenen et al. (2019). We tested for signatures of selection using CodeML, which is part of the PAML package version 4.9 (Yang 2007), implementing both the branch-site model (Zhang, Nielsen, and Yang 2005) and clade model C (Bielawski and Yang 2004). Phylogenetic trees for model fitting were created using the RAxML software version 8.2.12 on the CIPRES platform. Both branch-site and clade model C likelihoods were compared to respective null hypotheses using likelihood ratio tests (LRTs) and χ2 distributions. Bayes Empirical Bayes (Yang, Wong, and Nielsen 2005) inference was used to detect sites under positive selection. To test for rate variation at synonymous sites, we performed additional scans for selection in FUBAR (Murrell et al. 2013) and MEME (Murrell et al. 2012) in HYPHY (Pond , Frost, and Muse 2005) on the Datamonkey webserver (Delport et al. 2010). Posterior probabilities > 0.85 or *P* values < 0.1 were considered as evidence of positive selection for sites. RDP4 (Martin et al. 2015) was used with the concatenated genomes of all available TULV (Fig. 2) in order to detect reassortments and recombination. We used all methods available within the software to detect potential recombination events and only retained those which were detected across all methods to minimize uncertainty in recombination and breakpoint identification (Martin et al. 2017).

### Sequencing of host mitochondrial DNA

Common vole DNA was extracted according to a standard phenol–chloroform protocol. We used mtDNA for an initial assessment of the evolutionary lineages of voles across the Central transect. We sequenced at least 288 basepairs (bp) of the cytochrome *b* gene following Fink, Excoffier, and Heckel (2004), which allows unambiguous differentiation between mtDNA lineages (Sutter, Beysard, and Heckel 2013). We sequenced a total of 132 individuals, consisting of 119 new individuals from this study and thirteen additional ones from Saxenhofer et al. (2019) in the Porcelain transect, which have not been previously sequenced (Supplementary Table S1). We analysed at least two individuals per population whenever possible to obtain a general overview of host lineages across the entire Central transect. Mitochondrial lineages were assigned based on reference sequences from Braaker and Heckel (2009) (Supplementary Table S2). Phylogenetic analysis was performed in the same way as for the CDS of the TULV genomes (see earlier).

### Genotyping of host nuclear DNA

Genome-wide nuclear DNA (nucDNA) was used to infer the genetic structure of hosts via Genotyping by Sequencing (GBS) (Elshire et al. 2011). We sequenced at least five individuals per sampling site across the Central transect whenever possible, for a total of 200 individuals (Supplementary Table S1). Sequencing was carried out by LGC Biosearch Technologies (Berlin, Germany) using Illumina NovaSeq 6000 and PstI/MspI as restriction enzymes. We combined our dataset with GBS data of 216 additional individuals from the Porcelain transect (Saxenhofer et al. 2022) processed under the same conditions. This separate dataset consisted of voles from the Central and Eastern lineages, as well as admixed individuals, and served as a reference for the assignment of the newly genotyped 200 individuals to the evolutionary lineages.

SNP calling was performed for all 416 individuals together using the GBS v2 pipeline (Tassel 5) (Glaubitz et al. 2014) with the *M. arvalis* genome (BioProject ID: PRJNA737461, Gouy et al., submitted) as reference. We utilized default parameters, except requiring a minimum of five reads to identify a unique tag. We only retained bi-allelic SNPs and called genotypes if individuals had a read depth of at least five at the locus. After SNP calling, we removed all loci with complex indels, a minor allele frequency of less than 5 per cent, more than 20 per cent missing data or observed heterozygosity greater than 50 per cent, which may indicate loci that contain paralogues merged together (White et al. 2013). Individuals with more than 50 per cent missing data were also removed (seven individuals, all from Saxenhofer et al. (2022)). We performed a linkage disequilibrium k-nearest neighbors imputation in TASSEL 5 (Glaubitz et al. 2014) for remaining missing data based on the most common state of the allele across the ten closest genetic neighbours, calculated across the thirty SNPs with the highest LD towards the missing site and keeping Ns in the case of ties. A total of 12.8 per cent of data were missing in the dataset of 409 individuals, of those 99.93 per cent were imputed. Sites which still contained missing data after imputation were discarded. To address potential batch effects of combining two independent GBS datasets, six of the 200 sequenced individuals were replicates of samples from Saxenhofer et al. (2022). One of the replicates was among the seven samples which failed quality control, leaving a total of five effective replicates. We performed all analyses of host population structures with the original dataset before imputation, a second dataset after imputation of missing data, and a third, very stringently filtered dataset in which we removed any loci from our analysis at which SNPs differed between the originals and replicates. We found minor quantitative differences between the three datasets but identical qualitative patterns across all analyses and show only the results for the stringently filtered dataset.

### Host population structure

The genetic structure of common voles in the Central and Porcelain transect was analysed using the ADMIXTURE 1.3 software (Alexander, Novembre, and Lange 2009). Cross-validation (CV) was performed for cluster numbers from one through five, and CV error rates were used to determine the optimal number of clusters. ADMIXTURE was re-run for the optimal cluster number with 1,000 bootstrap replicates to establish cluster membership of all individuals. In addition, we performed a principal component analysis (PCA) using the SNPRelate package (Zheng et al. 2012) in R. For display, individuals from the Porcelain transect were assigned to evolutionary lineages based on cluster membership from the three-cluster model in admixture: Central lineage: *q*_cluster1_ + *q*_cluster2_ ≥ 0.9, Central–Eastern admixed: *q*_cluster1_ + *q*_cluster2_ ≥ 0.1 and *q*_cluster3_ ≥ 0.1, and Eastern lineage: *q*_cluster3_ ≥ 0.9.

We analysed genetic distances between host populations in order to compare the extent of population structures within the Central transect to the hybrid zone in the Porcelain transect. We first calculated pairwise *F*_ST_ (Weir and Cockerham 1984) between populations of four or more individuals within each transect with vcftools (Danecek et al. 2011). We then tested for isolation-by-distance (IBD) relationships between genetic and geographic distances among the populations within each transect via Mantel tests using the ecodist package (Goslee and Urban 2007) in R. We fitted linear models for each transect to test for differences in the slopes of regressions. We compared models with different slopes for each transect with a model with a single slope for both transects using a LRT in R.

### Cline analyses

In order to quantify the width and centre of the TULV clade contact zone and compare it to the genomic transition of host DNA in the Central transect, we performed an analysis of geographic clines using the HZAR package in R (Derryberry et al. 2014). We fitted a one-dimensional axis along the Central transect, which minimized the geographic distance between the TULV-CEN.N and TULV-CEN.S clades (Fig. 1). Sampling locations were projected onto the transect axis, and distances are given between the projection points (Beysard and Heckel 2014; Saxenhofer et al. 2019). For geographic cline fitting, we categorized each population based on its proportion of evolutionary lineages for mtDNA of the hosts, cluster memberships for nucDNA of the hosts, and the clade membership for TULV. For each of the different data types, four cline models were applied with increasing complexity of parameterization: null model (no cline within the sampling region), Model 1 (cline boundaries set to minimum and maximum observed frequency, free cline centre, and width), Model 2 (minimum and maximum frequency is free), and Model 3 (additional free parameters for independent exponential tails). We compared likelihood scores of all four cline models for each dataset and estimated cline parameters for the model with the highest likelihood, performing 10^5^ generations of MCMC sampling in three independent chains and with a burn-in period of 10^4^ iterations. Concordance of cline centres and widths was tested with a LRT in R. The test statistic was calculated as two times the difference between the log-likelihood of the alternative model of individual cline widths and centres for both datasets and a null model predicting a concordant cline through two combined datasets. Significance was determined based on a *χ*^2^ distribution with two degrees of freedom.

## Results

### TULV clade distribution and divergence

Screening of 247 adult voles for TULV RNA detected the S-segment fragment in forty-one individuals. We combined these with five TULV-positive samples from Saxenhofer et al. (2019), for which complete genomes are not yet available, for whole-genome sequencing. Forty-four of these samples passed library quality control, and hybrid sequence capture yielded a total of 2,409,747 TULV sequence reads (range: 4,435–222,961 per sample) that could be *de novo* assembled and backmapped. Read depth was very large across most of the forty-four sequenced genomes with an average of 960× (maximum: 3,103×, minimum: 31×; Supplementary Table S3). All genomes covered between 99.2 per cent and 100 per cent of the TULV ‘Moravia’ reference genome (Kukkonen, Vaheri, and Plyusnin 1998), and 99.25 per cent of all sites were covered by at least three reads and 96.4 per cent by at least twenty reads. Re-sequencing of a TULV sample (MarDHg01) from Saxenhofer et al. (2019) showed an identical sequence, albeit with a much larger depth (203× with sequence capture vs 31× with earlier shotgun sequencing).

Phylogenetic analysis assigned the forty-four new samples into twenty-three TULV-CEN.N and twenty-one TULV-CEN.S genomes both at the nucleotide and AA level (Fig. 2, Supplementary Fig. S1). Phylogenetic assignment of viral genomes to the two clades was consistent for all genomic segments. Our analyses with RDP4 indicated a potential reassortment event within the TULV-CEN.S clade (Supplementary Fig. S2) but no consistent evidence of recombination between the clades in any TULV genome segment. The newly sequenced TULV-CEN.N samples were found exclusively in the northern part of the Central transect and TULV-CEN.S in the southern part, without any discernible physical barriers (e.g. rivers, forests) to host dispersal or indications of lower host density between them (Fig. 1, Fig. 4B).

We included a published TULV-CEN.S genome (MarDSp01) from Saxenhofer et al. (2019) sampled in close proximity (6 km) to the Central transect in downstream analyses of the differences between the virus clades at the local geographic scale. Nucleotide divergence was 18 per cent between clades, while within-clade diversity was 6.9 per cent and 4 per cent for TULV-CEN.N and TULV-CEN.S, respectively (Table 1). Divergence in AA sequence was 3.25 per cent between clades and 0.57 per cent and 0.49 per cent within TULV-CEN.N and TULV-CEN.S, respectively. DEmARC analysis of the full AA sequence calculated PEDs at <0.04 and <0.01 between and within clades, respectively. Overall *d*_N_/*d*_S_ ratios were very similar between clades (0.011) and within TULV CEN.N (0.009) and TULV CEN.S (0.012). A sliding-window analysis showed that *d*_N_/*d*_S_ between the TULV-CEN.N and TULV-CEN.S clades was consistently low along the genome except for a *d*_N_/*d*_S_ spike at the beginning of the M-segment (Supplementary Fig. S3). Statistical support for positive selection was only provided by FUBAR for Codon 18 in this region of the M-segment (Supplementary Tables S4 and S5). All other statistical tests supported purifying selection along most of the TULV genome (Supplementary Tables S4 and S6). Our assessment of selective constraints across the phylogeny with clade Model C of CodeML confirmed purifying selection as the main selective force affecting 88.5–96 per cent of the viral CDS (Supplementary Table S5). However, the analysis also indicated divergent selective constraints between TULV-CEN.N and TULV-CEN.S particularly for the M-segment, with a significantly elevated proportion of sites under non-purifying/neutral selection compared to the null model (TULV-CEN.N: *P* = 0.023, TULV-CEN.S: *P* < 0.001; Supplementary Table S5). Additional positions of interest may include codons 644 and 764, both of which encode AAs that were fixed between the two clades and fell into different sidechain polarity groups, which may alter their binding and surface reactivity properties.

**Table 1.** Genome-wide sequence divergence of phylogenetic clades in TULV. The table shows the within-clade diversity of TULV-CEN.N (*n* = 23) and TULV-CEN.S (*n* = 22) and net divergence between the two clades across the Central transect. For each genome segment, the estimates are given for the CDS, the AA sequence, and *d*_N_/*d*_S_ ratios.

		TULV-CEN.N (*n* = 23)			TULV-CEN.S (*n* = 22)			Between clades		
Segment	Length	CDS (%)	AA (%)	*d*_N_/*d*_S_	CDS (%)	AA (%)	*d*_N_/*d*_S_	CDS (%)	AA (%)	*d*_N_/*d*_S_
L	6,459	8.82	0.76	0.009	3.12	0.40	0.012	18.54	3.67	0.007
M	3,423	4.65	0.41	0.009	6.10	0.79	0.014	18.12	2.29	0.009
S	1,287	3.91	0.04	0.004	2.62	0.15	0.005	14.88	3.67	0.023
All	11,169	6.92	0.57	0.009	3.98	0.49	0.012	17.99	3.25	0.011

### *Microtus arvalis* lineage distribution and population structure

**Figure 3.**
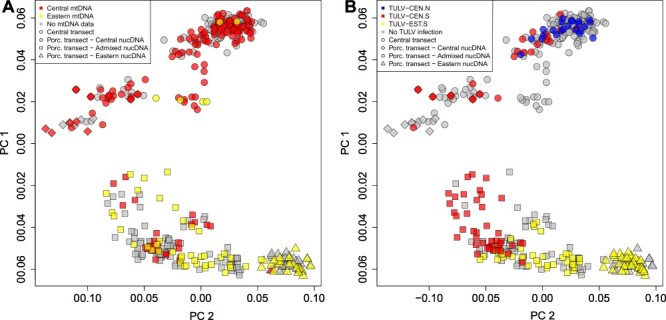
Associations of nuclear genomic variation in *M. arvalis* with (A) mitochondrial DNA and (B) TULV clades in the larger contact region between TULV clades. Both plots show the result of a PCA on nuclear SNPs in all 404 common voles. (A) Distribution of the Central (red) and Eastern (yellow) mitochondrial lineages in vole hosts. Grey: not sequenced for mitochondrial DNA. (B) Distribution of infections by TULV-CEN.N (blue), TULV-CEN.S (red) and TULV-EST.S (yellow) across all infected individuals. Grey: no TULV RNA detected. Principal component (PC)1 and PC2 explained 6.02 per cent and 1.53 per cent of total variance, respectively. Symbol shape indicates the transect of origin and lineage membership based on Fig. 3: circle: Central transect; rhombus: Porcelain transect, Central lineage; square: Porcelain transect, admixed between Central and Eastern lineage; and triangle: Eastern lineage.

Our survey of host mtDNA at forty-four sampling locations confirmed that the Central transect contained 114 Central and only five Eastern lineage sequences in the east in voles close to the hybrid zone (Fig. 1, Supplementary Fig. S4). For the nuclear genomes, the most stringently filtered final dataset consisted of 6,947 SNPs typed in 404 vole individuals (excluding technical replicates) across the Central and Porcelain transects. The PCA showed a population structure in the region, which resembles the actual geographical distribution of the samples and the presence of the Central and Eastern evolutionary lineages (Fig. 3). Individuals from the Central transect and the western part of the Porcelain transect were separated from the Eastern lineage part of the Porcelain transect, largely reflecting mtDNA lineage distributions (Fig. 3A). Continuous genomic transitions in host nucDNA in both transects were associated with largely discrete patterns of infection by the three TULV clades (Fig. 3B).

**Figure 4.**
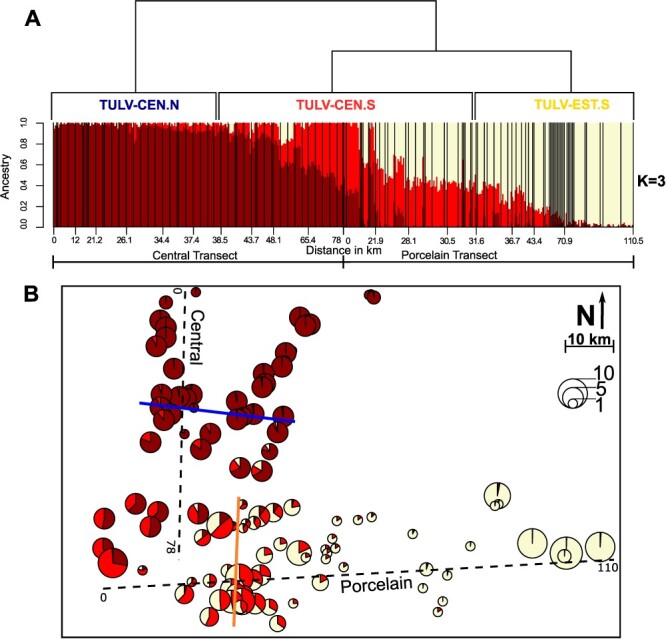
Genetic admixture of *M. arvalis* hosts in the larger contact region of TULV-CEN.N, TULV-CEN.S and TULV-EST.S. (A) Admixture analysis of nucDNA of 404 samples for *K* = 3. Each vertical bar represents the assignment of an individual to the genetic clusters (Cluster 1: dark red, Cluster 2: light red, and Cluster 3: light yellow). Geographical distances are given as the distance of the sampling site in kilometres from the respective transect start. Black vertical lines separate individuals from different sampling sites. The cladogram above the barplot indicates the phylogenetic relationships of the TULV-CEN.N, TULV-CEN.S and TULV-EST.S clades and their distribution along the two transects. (B) Spatial overview of cluster membership of voles for the three-cluster model (*K* = 3) from Admixture. Circle sizes are proportional to the number of individuals analysed per sampling site. The solid blue and orange lines show the location of contact between the TULV-CEN.N and TULV-CEN.S clades and TULV-CEN.S and TULV-EST.S clades, respectively.

Genetic clustering of nucDNA with ADMIXTURE revealed very similar support for three (CV error *K* = 3: 0.50142) and four genetic clusters (CV error *K* = 4: 0.50130) in our vole dataset. Partitioning into *K* = 3 showed a genomic and spatial transition between Cluster 1 in the northern part of the Central transect and Cluster 2 in the southern part (Fig. 4A). The large majority of voles infected with TULV-CEN.N or TULV-CEN.S in the Central transect contained mostly Cluster 1 ancestry. Cluster 2 showed further a gradual transition into Cluster 3 that was largely composed of individuals in the Porcelain transect assigned as Central–Eastern hybrids and Eastern lineage common voles in Saxenhofer et al. (2022). The gradual shift of ancestry toward the east within the Porcelain transect reflects the admixture zone between the host lineages and the abrupt transition between TULV-CEN.S and TULV-EST.S (Fig. 4B; Saxenhofer et al. 2019; Saxenhofer et al. 2022). Admixture analyses with *K* = 4 produced highly similar genomic and spatial patterns and assigned the fourth genetic cluster mostly to voles in the immediate zone of hybridization in the Porcelain transect (Supplementary Fig. S5).

**Figure 5.**
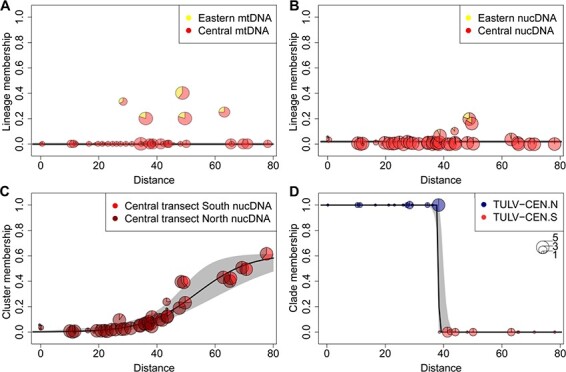
Geographical clines along the Central transect crossing the contact zone between the TULV-CEN.N and TULV-CEN.S clades. The transect was characterized based on the mtDNA of 119 voles (A), nucDNA of 200 voles (B and C), and clade assignment of forty-five TULV-infected voles (D). The *y*-axis shows the average membership towards the respective host lineages, genetic clusters, or TULV clades for each sampling site. In (A) and (B), the null model (no cline) had the lowest Akaike information criterion c (AICc). (C) shows an alternative membership assignment for nucDNA with a cline centre at 54.1 km and a cline width of 36.5 km. (D) The cline transition from TULV-CEN.N (blue) to TULV-CEN.S (red) occurred at 38.4 km with an estimated cline width of 0.007 km. 95 per cent credible cline regions are shown in grey. Distances are given relative to the northern end of the transect. Circle sizes correspond to sample sizes per site.

Direct comparisons of the spatial and genetic transitions between hosts and virus clades were performed using geographical cline analyses along the Central transect. For mtDNA, the cline null model was favoured consistent with no cline along the transect axis despite a few vole populations with introgressed Eastern lineage mtDNA (Fig. 5A; Supplementary Table S7). For nucDNA, combining admixture components from Clusters 1 and 2 vs. Cluster 3 also favoured the cline null model (Fig. 5B). The alternative combination of Cluster 1 vs. Clusters 2 and 3 supported a gradual transition along the Central transect (Fig. 5C) with the cline centre at 54.2 km (46.9–64.4 km 95 per cent confidence interval (CI)) and a width of 36.5 km (20.5–62.7 km 95 per cent CI). In contrast to the hosts, the clinal transition from the TULV-CEN.N to the TULV-CEN.S clade was extremely steep (Fig. 5D) with an estimated cline width of 0.0048 km (0.0003–1.3013 km 95 per cent CI) and the cline centre at 38.4 km (38.2–38.9 km 95 per cent CI). The TULV and host clines differed significantly in their centres and widths (LRT: *P* < 0.0001) (Supplementary Table S7).

**Figure 6.**
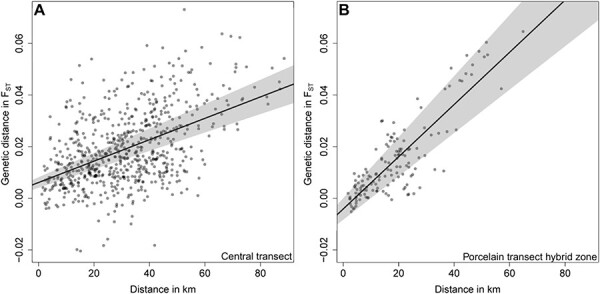
Genetic differentiation between common vole sampling locations in the Central and Porcelain transects. (A) Pairwise comparisons between locations along the Central transect. (B) Pairwise comparisons between locations along the Porcelain transect crossing the hybrid zone between the Central and Eastern evolutionary lineages in *M. arvalis*. (A) features a slope estimate of 0.000414, which is significantly lower (LRT: *P* < 0.0001) than the slope estimate of (B) at 0.00101. Pairwise *F*_ST_ was estimated between sampling sites comprising four or more individuals. 95 per cent confidence intervals around linear regression lines are plotted in grey.

Given largely gradual genomic transitions in common voles, we compared the extent of host population structures within the Central transect to that of the hybrid zone in the Porcelain transect. We found significant associations of genetic and spatial distances between populations (IBD) in the Central transect (Mantel test *R*^2^: 0.238, *P* < 0.0001) and in the Porcelain transect (Mantel test *R*^2^: 0.728, *P* < 0.0001; Fig. 6). However, the slope of the IBD relationship was significantly steeper (LRT: *P* < 0.0001) for populations across the hybrid zone between the Central and Eastern lineages (slope: 0.00101, Fig. 6B) than for vole populations in the Central transect (slope: 0.000414, Fig. 6A). This supported that the gradual genomic transition in the Central transect likely reflected the genetic structure between host populations of the same lineage.

## Discussion

Our study demonstrates genome-wide separation of two hantavirus clades occurring parapatrically in the common vole *M. arvalis*. The narrow viral clade transition without physical barriers to vole dispersal and thus also to virus transmission and the level of TULV divergence observed in our study are highly similar to a TULV contact zone, which is associated with two distinct evolutionary lineages in the vole host (Saxenhofer et al. 2019). These results suggest that viral divergence much below the level of officially named virus species can be ecologically and functionally highly relevant in natural populations.

### The virus clade transition and host population structure

Our analysis of common vole population structure showed a gradual genetic transition from north to south within the Central transect and low levels of admixture from the Eastern evolutionary lineage (Fig. 4). The small spatial scale of our study coupled with the absence of potential barriers to vole dispersal makes it unlikely that the population structure is strongly impacted by climatic differences (Gloria-Soria et al. 2017), landscape connectivity (Bastos-Silveira et al. 2012; Gryseels et al. 2017; Somoano et al. 2022), or other extrinsic factors. The genetic patterns in the Central transect are best explained by the population structure and IBD within a single evolutionary host lineage rather than by the presence of two host lineages (Fig. 5A and B).

IBD patterns have been documented in *M. arvalis* on large geographical scales (Heckel et al. 2005) as a consequence of the species’ limitation to short distances of dispersal and gene flow (Schweizer, Excoffier, and Heckel 2007; Hahne et al. 2011). Clustering algorithms like in the ADMIXTURE software sometimes tend to interpret IBD patterns as a transition between genetic clusters (Meirmans 2012) such as those inferred along our Central transect. Saxenhofer et al. (2019), (2022) classified the common voles in the area of Cluster 2 (Fig. 4B) as Central lineage, consistent with our mtDNA data. We cannot, however, fully exclude that Cluster 2 in our analysis may be associated with admixture between the Central and the Eastern evolutionary lineages. It is thus possible that introgression of specific alleles from the Eastern host lineage with barrier effects towards TULV-CEN.N could locally contribute to limiting the range of the clade (see later; Saxenhofer et al. 2022). However, the contact zone between TULV-CEN.N and TULV-CEN.S extends 500–600 km westward to the Netherlands entirely through the Central host lineage (Heyman et al. 2002; Heckel et al. 2005; Lischer, Excoffier, and Heckel 2014; Schmidt et al. 2016, 2021; Maas et al. 2017; Wang, Peischl, and Heckel 2023) (Fig. 1). It is thus unlikely that genetic variation specific to the Eastern lineage has an effect on the distribution of the TULV-CEN.N clade beyond host populations in close proximity to the Central–Eastern hybrid zone.

An alternative explanation for the transition of TULV clades along the Central transect would be their evolutionary association with an undetected sublineage within the Central host lineage. This scenario would require within the Central lineage a complete, large-scale replacement of mitochondrial DNA of one of the two sublineages and a shift of the TULV clade contact in our study area (Fig. 4A, Supplementary Table S7). Differences between the distribution of mtDNA lineages and nucDNA patterns have been observed in hybrid zones of several *Microtus* species including *M. arvalis* (Braaker and Heckel 2009; Bastos-Silveira et al. 2012; Beysard et al. 2012; Sutter, Beysard, and Heckel 2013; Beysard and Heckel 2014; Beysard, Krebs-Wheaton, and Heckel 2015). However, dense geographical coverage of genetic studies has provided no support for potential sublineages in the Central host lineage (Fink, Excoffier, and Heckel 2004; Heckel et al. 2005; Braaker and Heckel 2009; Martínková et al. 2013; Beysard and Heckel 2014; Fischer et al. 2014; Schmidt et al. 2016; Wang, Peischl, and Heckel 2023), making this evolutionary scenario unlikely.

### Evolutionary history of TULV clades and *M. arvalis* in Central Europe

The co-location of many borders between TULV clades and *M. arvalis* lineages (Fig. 1; Saxenhofer et al. 2019; Schmidt et al. 2016) suggests a major role of co-divergence processes in the evolutionary history of this system, similar to other hantaviruses and their hosts (Guo et al. 2013). A recent study suggested a potential origin of TULV in the Black Sea region based on partial S-segment sequence data (Cirkovic et al. 2023). The vast distribution range of TULV is only very sparsely sampled in the east (Cirkovic et al. 2023), but this would indicate that the Western and Central European TULV clades originated from a single ancestral strain from Eastern Europe similar to the deeper evolutionary history of *M. arvalis* (Heckel et al. 2005). The divergence of evolutionary lineages in *M. arvalis* is associated with the separation of populations in multiple refugia across Europe during the last glacial maximum (LGM) (Heckel et al. 2005; Lischer, Excoffier, and Heckel 2014), which probably also contributed to the separation of phylogeographic clades in TULV. The sister clades TULV-CEN.S and TULV-EST.S are likely the result of a host switch from Central to Eastern lineage hosts following the secondary contact of the two vole lineages in the hybrid zone much after the LGM (Beysard and Heckel 2014; Saxenhofer et al. 2019). The divergence between these clades has then been accumulated over time after being isolated in their respective host lineages.

The history of divergence is less clear for the more distant relationship of TULV-CEN.N and TULV-CEN.S (Fig. 2) given the lack of genomes from other European clades, e.g. TULV Eastern North (TULV-EST.N) in the northern part of the Eastern host lineage. Partial genome sequence data exist but provide no robust support for the more basal nodes in phylogenetic reconstructions of TULV (e.g. Saxenhofer et al. 2017, 2019; Schmidt et al. 2021; Cirkovic et al. 2023). The wider application of sequence capture methods such as the one used here has the potential to provide much more resolution of (co-)evolutionary processes in TULV and other pathogens in the future (e.g. Hiltbrunner and Heckel 2020; Jeske et al. 2021).

### Genome-wide isolation and incipient species in TULV

The absence of detectable recombination or reassortment between TULV-CEN.N and TULV-CEN.S in our study indicates distinct species according to the concept for acellular organisms of Bobay and Ochman (2018). However, due to their low divergence at the AA level and several other criteria, they do not meet the ICTV thresholds for distinct virus species. Rather, they can be classified as incipient species, given that barriers to genetic exchange are apparently already in effect. In general, reassortment events in hantaviruses have been observed only infrequently and all documented cases occurred within ICTV-recognized species (Klempa 2018). Co-infection with two different TULV clades, as a necessary prerequisite for reassortment or recombination to occur, has been detected only in a few individuals at the contact between TULV-CEN.S and TULV-EST.S (Saxenhofer et al. 2019; Hiltbrunner and Heckel 2020), but not in the present study. TULV prevalence generally varies between 0 and 58 per cent across populations, with an average of around 15–20 per cent in adult voles (Schmidt-Chanasit et al. 2010; Schmidt et al. 2016, 2021; Maas et al. 2017; Jeske et al. 2021). Moderate rates of infection among both dispersing and local vole individuals are therefore likely to limit the potential for double infections with two viral clades. Potential traces of older recombination events within TULV clades were detected in a few sequences, but no evidence of recombination between clades has been found (Saxenhofer et al. 2019; Hiltbrunner and Heckel 2020).

Reassortments between TULV-CEN.N and TULV-CEN.S may occasionally happen, because two potentially reassorted samples were detected in a population about 360 km to the west of our study area (Schmidt et al. 2021). The inference suggesting likely reassortment was based only on partial TULV genome sequences, making the final distinction from recombination or co-infection impossible. Given the relatively dense sampling of TULV diversity in the studies of clade contact zones here and in Saxenhofer et al. (2019), we suggest that the products of reassortment or recombination between clades are probably less fit than their ancestral counterparts and get purged from populations (see e.g. McDonald et al. 2016).

### Adaptive interactions between TULV and vole genomes

Genome-wide data have suggested a particular adaptive role for clade-specific differences in a set of five codons at the beginning of the TULV M-segment in this study for TULV-CEN.N and TULV-CEN.S and Saxenhofer et al. (2019) for TULV CEN.S and TULV-EST.S. This region corresponds to the N-terminus of the glycoprotein and may represent a signal peptide or the beginning of the N-terminal ectodomain (Vaheri et al. 2013; Ganaie and Mir 2014; Li et al. 2016). Interactions between this region and specific host genes are hypothesized to be drivers of TULV diversification and speciation (Saxenhofer et al. 2019, 2022). In particular, Codon 17 of the M-segment of TULV-CEN.S and TULV-EST.S was found to be under positive selection (Saxenhofer et al. 2019) and our analyses here showed indications for Codon 18. The more than five-fold increase in *d*_N_/*d*_S_ at the beginning of the M-segment compared to the rest of the genome (Supplementary Fig. S3) suggests differences in the adaptive regime. However, high rates of synonymous variation and purifying selection on linked sites may prevent the detection of an even stronger signature of positive selection.

On the host side, several candidate genes with a potential effect as a barrier to the transmission of non-adapted TULV-CEN.S or TULV-EST.S clades have been identified in a genome-wide association study (Saxenhofer et al. 2022). These genes may be targets for interactions with TULV in general and the region at the beginning of the M-segment in particular. However, most of the SNP alleles significantly associated with infection by TULV-CEN.S or TULV-EST.S in the hybrid zone between evolutionary lineages were not present in the Central transect dataset. The sharp parapatric distribution of the TULV-CEN.N and TULV-CEN.S clades is nevertheless likely to be associated with yet undefined genomic barriers in the vole host. It is possible that the same genome region in different TULV clades may interact differently with multiple genomic regions in the common vole hosts depending on their deeper evolutionary background. An analogous system was described for the 3ʹ untranslated region in the Dengue virus and its mosquito and human hosts (Villordo et al. 2015). The viral region has specific adaptations to each host, potentially suppressing Interferon-α/β activities in human cells and RNAi pathways in mosquito cells (Villordo et al. 2015). Extended genomic and functional analyses will be necessary in the future to determine the general and specific interactions between TULV clades and their hosts.

### Evolving virus species in single host species

The generation of new species in parasites in general and viruses in particular is most commonly studied in the context of host–parasite co-divergence and host shifts (Switzer et al. 2005; Rector et al. 2007; Parrish et al. 2008; Sharp and Simmonds 2011; Lin et al. 2012; Longdon et al. 2014; Mélade et al. 2016). Speciation or co-existence of sibling parasite species within single host species has only been documented in a few cases and requires specific conditions, e.g. within species variation of key host genes (Pérez-Tris et al. 2007; Meyer et al. 2016; Martinů, Hypša, and Štefka 2018; Saxenhofer et al. 2019; Martinů et al. 2020; Chaikeeratisak et al. 2021). Viral contact zones within a single reservoir host can provide important insights into the genetic environment that can facilitate speciation and the extent of functional divergence between incipient species. For example, the contacts between parapatric arenaviruses within morphologically cryptic evolutionary lineages of their rodent host show similarities with the TULV case (Gryseels et al. 2017; Cuypers et al. 2020) even though lower spatial and genetic resolution limits the conclusions. In comparison, murine cytomegalovirus shows multiple viral clades that are associated with *Mus musculus musculus* or *M. m. domesticus* in the European hybrid zone, but lacks the sharp contact (de Bellocq et al. 2015). The murine cytomegalovirus genome has been shaped by extensive recombination (Smith et al. 2013), which indicates that its clades belong to the same virus species despite association with separate host species. These findings emphasize that host divergence is not necessarily a direct indicator for virus divergence in closely related species and highlight the importance of high-resolution studies for the discovery and characterization of barriers to viral transmission in genetically similar hosts.

## Conclusions

The diversification of viruses within and between host species involves complex processes, which are vital for our understanding of the evolution and management of viruses around the globe. The combined analysis of virus and host genomes allowed us to determine that parapatric TULV clades behave from an evolutionary point of view like incipient species within a highly similar genetic environment. A particular feature of this system is that the multiple levels of divergence in both the virus and the host enable further studies on hierarchical phylogenetic levels and thus have the potential to provide much deeper insights than systems with one level only. Further studies support the idea that small genetic differences not only between viruses but also between hosts can be crucial for limiting the infection ranges of viruses (Stremlau et al. 2004; van Doremalen et al. 2014; Long et al. 2016). The partial decoupling of virus evolution from relatively simple co-divergence processes stresses the importance of combining information on both agents in host–parasite systems. We expect that in-depth characterization and phylogenomic analyses of pathogen populations together with their reservoir hosts will contribute to uncovering the full spectrum of factors that constitute fundamental barriers to viral transmission in genetically highly similar hosts and ultimately drive viral speciation.

## Supplementary Material

veae002_Supp

## Data Availability

Tula Hantavirus S-segment and common vole mtDNA sequences: National Center for Biotechnology Information GenBank accession numbers OP173222-OP173485. Genotype by Sequencing data: Sequence read archive accession number: PRJNA869681. Keyfile for Genotype by Sequencing data: Dryad repository DOI: 10.5061/dryad.w0vt4b905.
