# Supplementary material for: Genome-wide support for incipient Tula hantavirus species within a single rodent host lineage
Source: Virus Evol. 2024 Jan 5;10(1):veae002. doi: 10.1093/ve/veae002 (PMC10868551; doi:10.1093/ve/veae002)
Supplement: veae002_Supp [file veae002_supp.zip › suppl_data/Labutin&Heckel_VirusEvolution_SupInfo_final.docx]

**Supplemental Information for:**

**Genome-wide support for incipient Tula hantavirus species within a single rodent host lineage**

Anton Labutin, Gerald Heckel


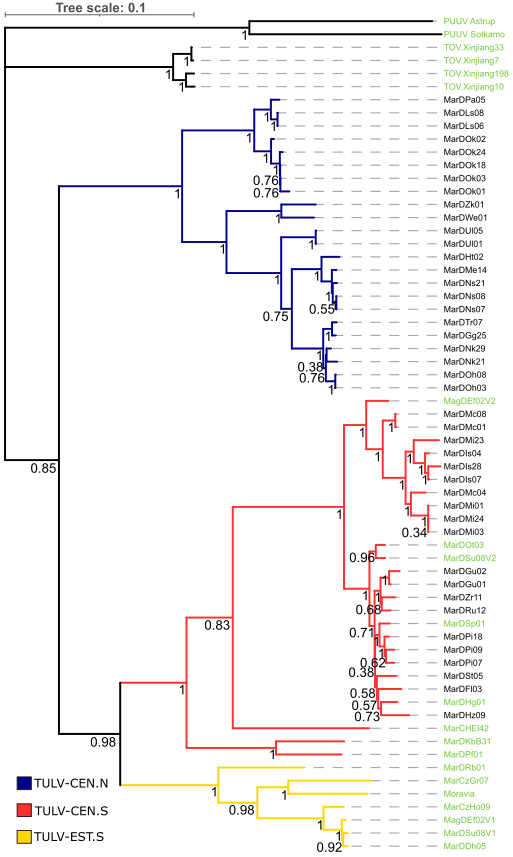


**Supplementary Figure 1. Phylogenetic relationships of all published TULV genomes with complete amino acid sequences.** Phylogenetic analysis was based on the concatenated amino acid sequences of TULV with Puumala orthohantvirus (PUUV) as outgroup. Names in black show new TULV genome sequences from this study while names in green represent references sequences. Bootstrap support values (1000 replicates) are included for all nodes. The scale bar on top shows evolutionary distance in substitutions per amino acid.


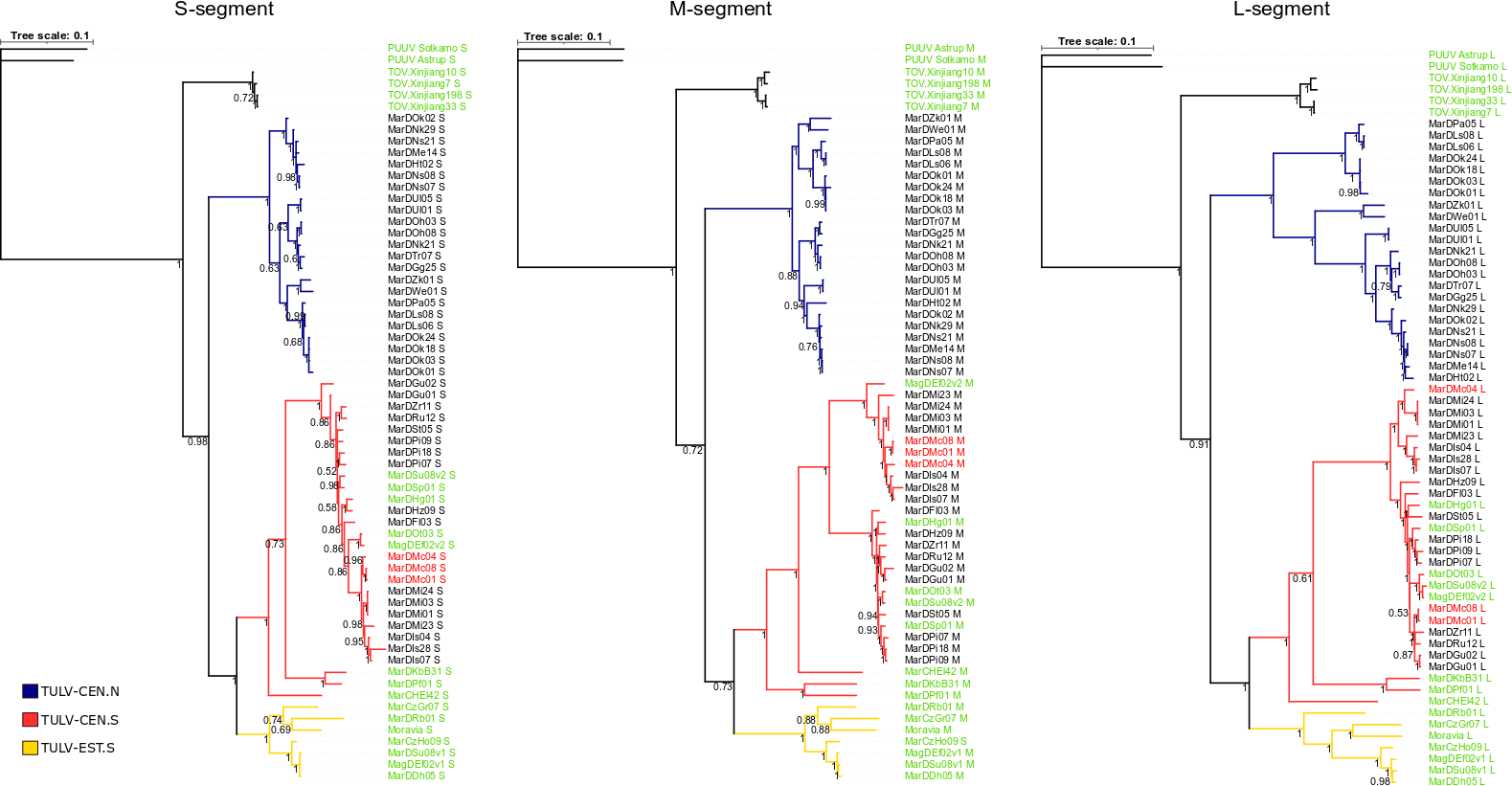


**Supplementary Figure 2. Phylogenetic relationships of all published TULV genomes with complete coding sequences.** Phylogenetic analysis of the S, M and L segment without imputation from TULV with PUUV as outgroup. Names in black show new TULV genome sequences from this study. Names in green represent reference and outgroup sequences. Names in red represent sequences from a TULV-CEN.S sampling site for which a reassortment event was observed in the L-Segment of MarDMc01 and MarDMc08. Bayesian posterior probabilities are included for all nodes. The scale bar on top shows evolutionary distance in substitutions per nucleotide.


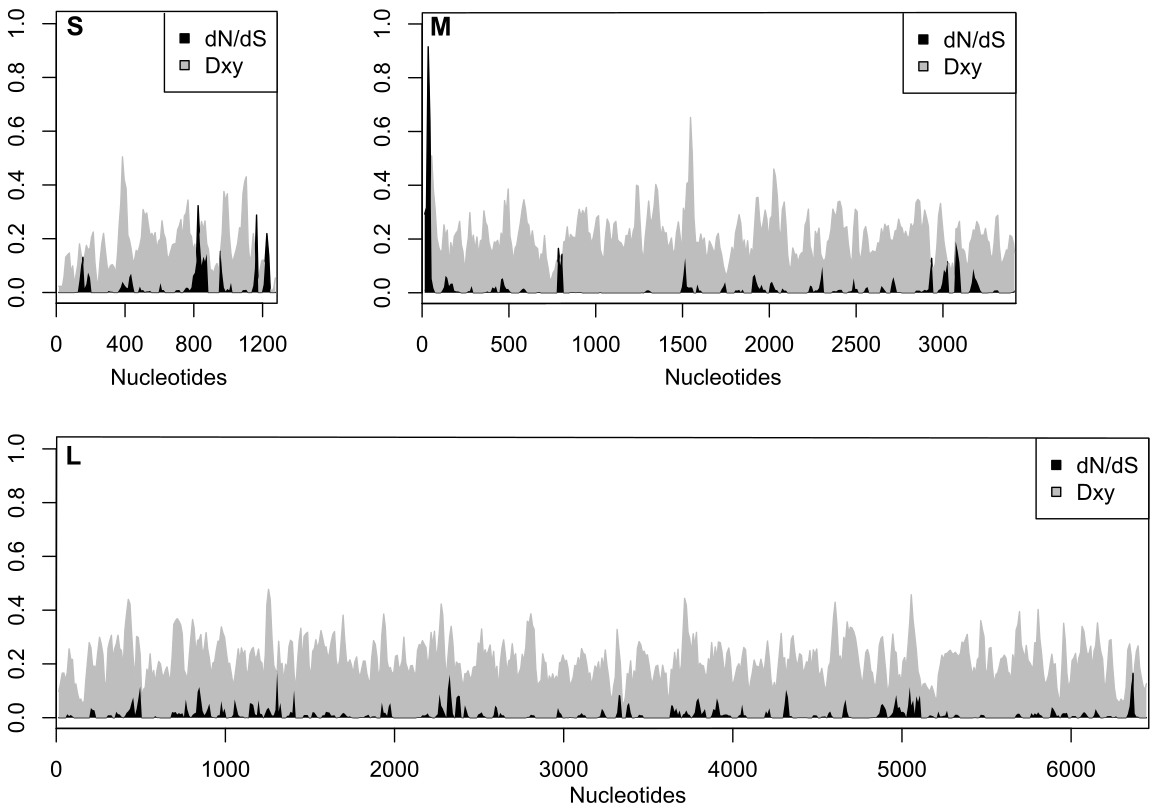
 **Supplementary Figure 3: Sliding window analysis of the TULV L, M & S segments**. The plots show the ratio of non-synonymous to synonymous substitutions (d_N_/d_S_, black area) and average number of nucleotide substitutions per site (D_XY_, grey area) between 23 TULV-CEN.N genomes and 22 TULV-CEN.S genomes. Only the coding sequence of the TULV segments was considered for the analyses. The window size was 30 nt and step size 10 nt.


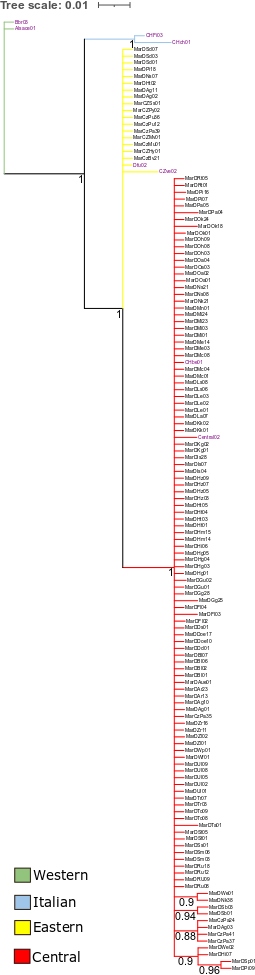


**Supplementary Figure 4. Phylogenetic clustering and lineage assignment of vole mitochondrial DNA.** The phylogenetic analysis was based on 288 bp of the cytochrome *b* gene. Names in purple represent reference sequences for the four major evolutionary lineages of *Microtus arvalis* in Europe from Braaker and Heckel (2009). Bayesian posterior probabilities are included for all nodes. The scale bar on top shows evolutionary distance in substitutions per nucleotide.


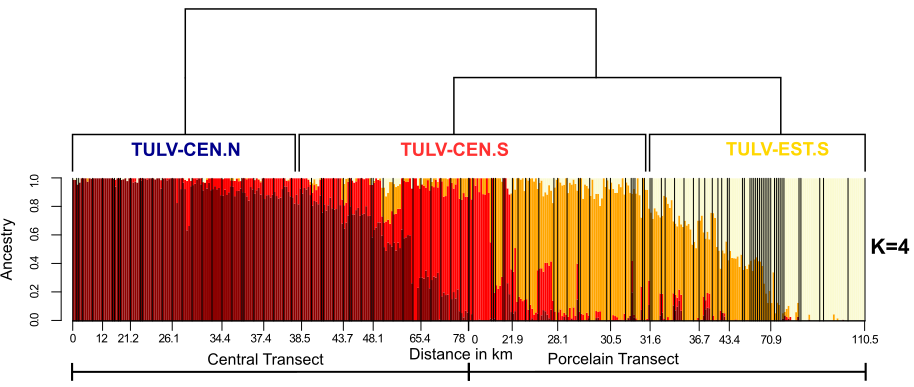


**Supplementary Figure 5. Alternative cluster model for the genetic admixture of common vole hosts in the contact region of TULV-CEN.N, TULV-CEN.S and TULV-EST.S.** Admixture analysis of nucDNA of 404 samples for K = 4. Each vertical bar represents the assignment of an individual to the genetic clusters (Cluster 1: dark red, Cluster 2: light red, Cluster 3: orange, Cluster 4: light yellow). Geographical distances are given as the distance of the sampling site in km from the respective transect start. Black vertical lines separate individuals from different sampling sites. The cladogram above the barplot indicates the phylogenetic relationships of the TULV-CEN.N, TULV-CEN.S and TULV-EST.S clades along the two transects.

**Supplementary Table 1: Overview over all common voles sampled for this study.** The Cytochrome *b* columns shows the vole’s mitochondrial lineage. The TULV genome column shows the TULV clade of infected individuals based on sequencing of the whole viral genome. The GBS column shows which common voles have GBS data available.

**Supplementary Table 2:** **Obtained sequences for phylogenetic clustering of TULV and mtDNA.** Listed sequences were collected from the NCBI database and used for the assignment of clades to phylogenetic clusters of TULV.

| f | Accession number | Sequence Title | Clade |
| --- | --- | --- | --- |
| S | NC005224 | PUUV Sotkamo | PUUV |
| S | KJ994776 | PUUV Astrup | PUUV |
| S | MN052670 | TOV.Xinjiang7 | Chinese outgroup |
| S | MN052671 | TOV.Xinjiang10 | Chinese outgroup |
| S | MN052672 | TOV.Xinjiang33 | Chinese outgroup |
| S | MN052673 | TOV.Xinjiang198 | Chinese outgroup |
| S | MT514276 | MagDEf02V2 | Central South |
| S | MK386134 | MarDOt03 | Central South |
| S | MT514280 | MarDSu08V2 | Central South |
| S | MK386135 | MarDSp01 | Central South |
| S | MK386133 | MarDHg01 | Central South |
| S | MT514277 | MarCHEl42 | Central South |
| S | MK386138 | MarDKbB31 | Central South |
| S | MK386139 | MarDPf01 | Central South |
| S | MK386140 | MarDRb01 | Eastern South |
| S | MT514278 | MarCzGr07 | Eastern South |
| S | Z69991 | Moravia | Eastern South |
| S | MK386131 | MarCzHo09 | Eastern South |
| S | MT514275 | MagDEf02V1 | Eastern South |
| S | MT514279 | MarDSu08V1 | Eastern South |
| S | MK386130 | MarDDh05 | Eastern South |
| M | NC005223 | PUUV Sotkamo | PUUV |
| M | KJ994777 | PUUV Astrup | PUUV |
| M | MN183137 | TOV.Xinjiang7 | Chinese outgroup |
| M | MN183138 | TOV.Xinjiang10 | Chinese outgroup |
| M | MN183139 | TOV.Xinjiang33 | Chinese outgroup |
| M | MN183140 | TOV.Xinjiang198 | Chinese outgroup |
| M | MT514286 | MagDEf02V2 | Central South |
| M | MK386146 | MarDOt03 | Central South |
| M | MT514290 | MarDSu08V2 | Central South |
| M | MK386147 | MarDSp01 | Central South |
| M | MK386145 | MarDHg01 | Central South |
| M | MT514287 | MarCHEl42 | Central South |
| M | MK386150 | MarDKbB31 | Central South |
| M | MK386151 | MarDPf01 | Central South |
| M | MK386152 | MarDRb01 | Eastern South |
| M | MT514288 | MarCzGr07 | Eastern South |
| M | Z69993 | Moravia | Eastern South |
| M | MK386143 | MarCzHo09 | Eastern South |
| M | MT514285 | MagDEf02V1 | Eastern South |
| M | MT514289 | MarDSu08V1 | Eastern South |
| M | MK386142 | MarDDh05 | Eastern South |
| L | KJ994778 | PUUV Astrup | PUUV |
| L | NC005225 | PUUV Sotkamo | PUUV |
| L | MN183133 | TOV.Xinjiang7 | Chinese outgroup |
| L | MN183134 | TOV.Xinjiang10 | Chinese outgroup |
| L | MN183135 | TOV.Xinjiang33 | Chinese outgroup |
| L | MN183136 | TOV.Xinjiang198 | Chinese outgroup |
| L | MT514296 | MagDEf02V2 | Central South |
| L | MK386158 | MarDOt03 | Central South |
| L | MT514300 | MarDSu08V2 | Central South |
| L | MK386159 | MarDSp01 | Central South |
| L | MK386157 | MarDHg01 | Central South |
| L | MT514297 | MarCHEl42 | Central South |
| L | MK386162 | MarDKbB31 | Central South |
| L | MK386163 | MarDPf01 | Central South |
| L | MK386164 | MarDRb01 | Eastern South |
| L | MT514298 | MarCzGr07 | Eastern South |
| L | AJ005637 | Moravia | Eastern South |
| L | MK386155 | MarCzHo09 | Eastern South |
| L | MT514295 | MagDEf02V1 | Eastern South |
| L | MT514299 | MarDSu08V1 | Eastern South |
| L | MK386154 | MarDDh05 | Eastern South |
| Cyt. b | AY708510 | Bbr03 | Western |
| Cyt. b | FJ789987 | Alsace01 | Western |
| Cyt. b | FJ789998 | CHFi03 | Italian |
| Cyt. b | AY708512 | CHch01 | Italian |
| Cyt. b | AY708475 | Dfu02 | Eastern |
| Cyt. b | AY708472 | CZve02 | Eastern |
| Cyt. b | AY708463 | CHbe01 | Central |
| Cyt. b | FJ789989 | Central02 | Central |

**Supplementary Table 3: Coverage statistics for all sequenced TULV genomes.** The table is subdivided into metrics for the CDS of each of the three TULV segments L, M & S, as well as for the combined coding sequence of the TULV genome. In each case, the table shows the length of the CDS, total number of assembled reads, average read depth, the percentage of sites with a read depth of at least 3 and the percentage of all sites with a read depth of at least 20.

**Supplementary Table 4: Results of the analysis for signatures of selection with the branch-site model for the TULV L, M & S Segments.** The branch site (BrS) test was performed in CodeML, which is part of the PAML package. The data was partitioned into the TULV-CEN.N and TULV-CEN.S clades. Each clade was analysed once as foreground branch with the other clade set to the background branch. For the BrS model CodeML was set to model=2 and NSsites=2 and in each case an estimated ω2 was compared against the BrS_null model in which ω2 in the foreground branches was set to 1. Codons detected as positively selected by Bayes empirical bayes inference are indicated with their posterior probability. Abbreviations in the table read as follows: np, number of model parameters; lnL, model likelihood; κ, transition to transversion ratio; ω, d_N_/d_S_ ratio, LRT, the D value of a likelihood ratio test; p-value, the p-value derived from a χ^2^ distribution with 1 degree of freedom.

**Supplementary Table 5: Results of the analysis for signatures of selection with the Clade Model C for the TULV L, M & S Segments.** The clade model C (CmC) test was performed in CodeML, which is part of the PAML package. The data was partitioned into the TULV-CEN.N and TULV-CEN.S clades. Each clade was analysed once as foreground branch with the other clade set to the background branch. For the CmC model CodeML was set to model=3 and NSsites=2, which estimates a separate ω for each of the clades. The results were in each case compared against the CmC_null model M2_rel in which the model was set to 0 and NSsites was set to 22, in which ω is fixed among clades. No codons were detected to be positively selected by Bayes empirical bayes inference. Abbreviations in the table read as follows: np, number of model parameters; lnL, model likelihood; κ, transition to transversion ratio; ω, d_N_/d_S_ ratio, LRT, the D value of a likelihood ratio test; p-value, the p-value derived from a χ^2^ distribution with 1 degree of freedom.

**Supplementary Table 6: Results of the analysis for signatures of selection with the MEME and FUBAR methods in HYPHY for the TULV L, M & S Segments.** The methods were used to detect amino acids evolving under positive or purifying selection. Positively selected codons are indicated with the posterior probability P for FUBAR or a p-value for MEME.

**Supplementary Table 7: Results of the cline analyses for the Central transect**. The table shows the Akaike information criterion (AICc) for each of the four models implemented in hzar. Lower scores indicate the highest level of support for a particular cline model.
